# A Miniature Resonant and Torsional Magnetometer Based on Lorentz Force

**DOI:** 10.3390/mi9120666

**Published:** 2018-12-17

**Authors:** Lingqi Wu, Zheng Tian, Dahai Ren, Zheng You

**Affiliations:** State Key Laboratory of Precision Measurement Technology and Instruments, Department of Precision Instrument, Tsinghua University, Beijing 100084, China; wlq03@mails.tsinghua.edu.cn (L.W.); tz13@tsinghua.org.cn (Z.T.); yz-dpi@tsinghua.edu.cn (Z.Y.)

**Keywords:** magnetometer, MEMS, resonance, Lorentz force, attitude determination

## Abstract

A microelectromechanical system (MEMS) torsional resonant magnetometer based on Lorentz force was investigated, consisting of torsional structures, torsional beams, metal plates, a coil, and a glass substrate. The Lorentz force, introduced by the interaction between the current in the MEMS coil and an external horizontal magnetic field, leads to displacement of the torsional structure. The strength of the magnetic field is proportional to this displacement, and can be detected with two sensing capacitors fabricated on the torsion structure and the substrate. To improve sensor sensitivity, a folded torsional beam and a double-layer excitation coil were introduced. The fabrication processes included lift-off, anodic bonding, chemical mechanical planarization, silicon nitride (SiNx) deposition, plasma-enhanced chemical vapor deposition, and inductively coupled plasma release. The prototype of the magnetometer was finished and packaged. The sensor performance, including its sensitivity and repeatability, was tested in a low-pressure environment. Additionally, the influences of structural parameters were analyzed, including the resistance of the excitation coil, the initial value of the capacitors, the elastic coefficient of the torsional beam, and the number of layers in the excitation coil. The test results demonstrated that this sensor could meet the requirements for attitude determination systems in low earth orbit satellites.

## 1. Introduction

Magnetometers are widely employed in geodesic surveys and aircraft attitude control systems as geomagnetic field detecting sensors [1,2,3]. As one of the most critical components in the attitude determination systems of spacecraft, they need to be both highly sensitive and accurate. However, the measurement range of the magnetometers can be less than that of sensors used on earth [4,5,6,7]. Additionally, miniaturization is important for the application of magnetometers in small satellites [8,9,10,11]. Compared to microelectromechanical system (MEMS) magnetometers based on the fluxgate effect [12,13], Hall effect [14,15], and magnetoresistance [16,17], microtorsional resonant magnetometers based on the Lorentz force have the advantages of a high quality factor and high reliability [18,19,20,21]. Meanwhile, its MEMS fabrication process is relatively simple [22,23]. Many Lorentz force-based sensors have high requirements for the sensitivity of capacitance measurement, which must be better than 10 aF [24,25,26]. Combining this with new fabrication methods or other sensors, more new devices were developed [27,28]. In this paper, a MEMS torsional resonant magnetometer based on the Lorentz force was studied, and the relevant parameters were optimized.

## 2. Principles and Simulations

**Figure 1:**
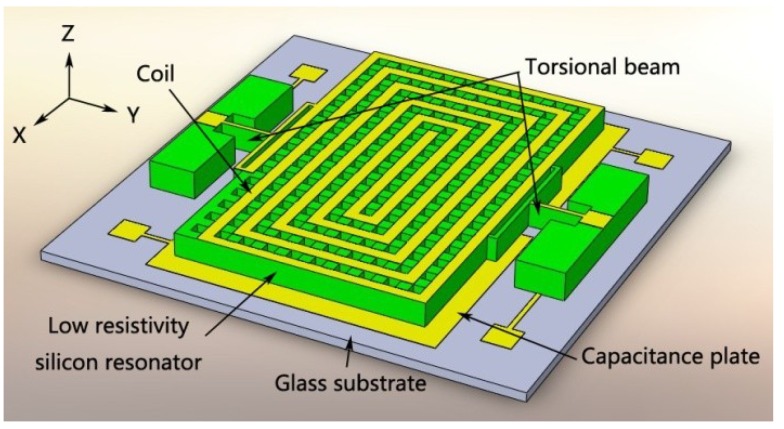
Principle of the microelectromechanical system (MEMS) torsional resonant magnetometer.

The MEMS structures of the magnetometer include torsional structures, torsional beams, metal plates, a coil, and a glass substrate (Figure 1). The Lorentz force is introduced by the current in the coil and the external horizontal magnetic field, and leads to differential displacement of the torsional structure, which can be detected with two sensing capacitors fabricated on the torsional structure. The torsion is along the *y* axis. All the rotation direction conforms to the right-hand rule.

Assuming that *k* is the elastic coefficient of the torsional beam, and *Θ* is half the rotational inertia of the torsional structures, then after derivation, the intrinsic frequency and critical damping of the resonance system could be obtained as follows:

(1) $$\omega =\sqrt{k/\Theta },$$

(2) c=2Θαω,

where *α* is the damping ratio of the torsional system; *k* is a function of the length, width of the torsional beam, and the width and height of the torsional structure; and *Θ* is a function of the above parameters and the density of the torsional beam material.

To increase the sensitivity of the sensor, a structure of folded torsional beams and a double-layer excitation coil was designed based on simulation and testing results for the structures of a preliminary fabrication. The structural parameters were also optimized. 

**Figure 2:**
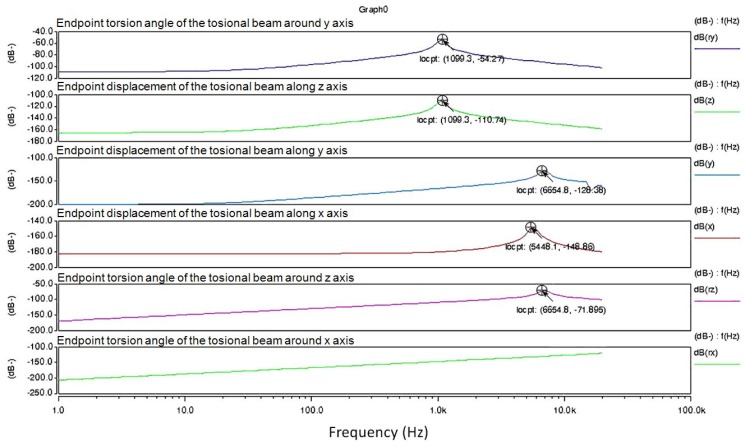
Simulation results for the resonant frequencies of the magnetometer in different directions, indicating the endpoint displacement of the torsional beams along three perpendicular directions and around three axes. Reproduced with permission from [29], published by IEEE, 2013.

Based on the principle and structure of the sensor, maximum system sensitivity can be achieved when the excitation signal frequency is in accordance with the resonance frequency of the torsional system. Using the small signal alternating current (AC) sweep analysis in the Architect module of CoventorWare, the endpoint displacement of the torsional beams was obtained along three perpendicular directions and around three axes (Figure 2). It can be seen that the resonant frequencies in the *z* and *ry* directions were very similar (about 1100 Hz). However, the resonant frequencies in the *x*, *y*, and *rz* directions were approximately 5 kHz to 6 kHz, which is far from 1100 Hz. With appropriate excitation signal frequencies, the vibrations in these directions could be suppressed. There was no peak in the vibration in the *rx* direction. When the frequency was less than 1100 Hz, its attenuation was approximately −150 dB.

Simulations indicated that the resonant frequencies in the *ry* and *z* directions were roughly the same, and there was no resonance peak in the *rx* direction. This is because its endpoint also vibrated in the *z* direction (vertical movement) when the torsional pendulum vibrated in the *ry* direction (around the *y* axis). The intensity in *ry* was approximately −54.3 dB at 1100 Hz, and the vibrating amplitude in the *rx* direction was approximately −150 dB, which was far less than that of the *ry* direction. Therefore, the torsional vibration around the *y* axis achieved its maximum intensity when the excitation signal frequency was the same as the resonant frequency of *ry*. However, the vibration amplitudes in the other directions were far less than those in this direction. From the perspective of the resonant frequency, these results demonstrated the rationality of the structure design.

## 3. Fabrication

The MEMS fabrication process included dry etching, a lift-off process, anodic bonding, chemical mechanical planarization (CMP), physical vapor deposition, electroplating, and inductively coupled plasma (ICP) etching. The materials used in the microfabrication process were a Pyrex glass substrate and a low-resistivity silicon wafer with a diameter of 4 inches and a thickness of 200 μm. 

**Figure 3:**
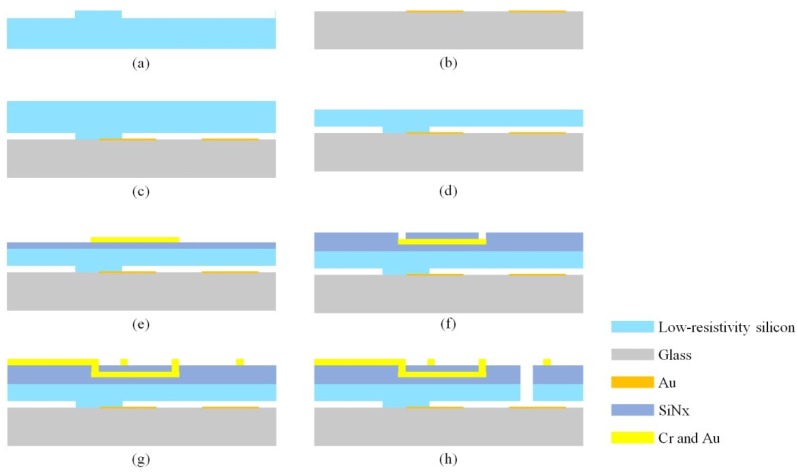
Fabrication process for the MEMS structures: (**a**) A step was etched on the backside of the low-resistivity silicon for glass–silicon bonding. (**b**) Fabrication of the metal plates on the glass substrate with the lift-off process. (**c**) The low-resistivity silicon was attached to the glass substrate through anodic bonding. (**d**) The silicon wafer was polished using chemical mechanical planarization (CMP). (**e**) Gold was patterned to fabricate the lead wire. (**f**) Silicon nitride (SiNx) was deposited for insulation. (**g**) Gold was patterned to fabricate the coil layer using plasma-enhanced chemical vapor deposition (PECVD). (**h**) The torsional structures were released with inductively coupled plasma (ICP).

The optimized fabrication process is shown in Figure 3. First, a step was etched on the backside of the low-resistivity silicon for glass–silicon bonding, as shown in Figure 3a. Then, the metal plates were fabricated on the glass substrate with the lift-off process, as shown in Figure 3b. Subsequently, a low-resistivity silicon wafer was attached to the glass substrate through anodic bonding (Figure 3c), and the silicon wafer was polished using CMP (Figure 3d). Then, silicon nitride (SiNx) was deposited for insulation (Figure 3e), and gold was patterned to fabricate the coil layer using plasma-enhanced chemical vapor deposition (PECVD) (Figure 3f–g). Finally, the torsional structures were released with ICP, as shown in Figure 3h.

Based on preliminary fabrications and tests, the thickness of the low-resistivity silicon wafer was further decreased to 60 µm with CMP before depositing the SiNx insulating layer. This improvement greatly enlarged the torsional angle and increased the convenience of the subsequent ICP process. Thinning the structure layer effectively increased the success rate of the ICP and ensured good mechanical properties of the torsional beam. However, it also created undesirable structural risks. For example, the back of the silicon wafer has a step structure, which forms a cavity with the glass substrate. The pressure on the silicon wafer during the CMP process may break this structure. Therefore, this cavity area should be reduced to prevent the silicon wafer from being crushed. 

To reduce the silicon–gold contact resistance introduced by the bonding process, an etching process was added before silicon–glass bonding to remove the oxide layer on the low-resistivity silicon. With this additional process, the resistance of the torsional structure was approximately 1 kΩ, which was very close to the simulation result. By peeling the torsional structure from the device and measuring it with a probe, its resistance was found to be only 500 Ω. This indicates that the silicon–gold contact resistance was reduced to approximately 500 Ω. The oxide film etching process produced low-resistivity silicon directly bonded to the gold material, avoiding reduction in the conductivity caused by the oxide layer on the silicon. This benefited the subsequent high-precision measurement of the capacitance.

To increase the sensitivity, the torsional beams of the device were designed as folded beams to reduce the elastic coefficient. In addition, two coil layers were achieved in the devices, which greatly increased the sensitivity without increasing the fabrication difficulty.

**Figure 4:**
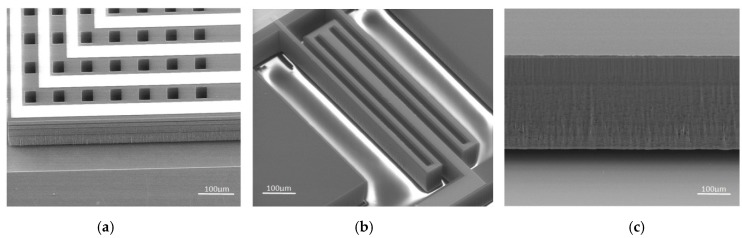
Fabrication results for the (**a**) coil, (**b**) folded beam, and (**c**) sidewall.

Figure 4 shows the fabrication results for the key structures. It can be seen that the folded beam structure was intact, and the sidewall of the torsional structures and coil were of good quality. Damping holes were released completely by ICP, reducing the squeeze film air damping effect. These results indicate that by reducing the thickness of the structural layer, both the fabrication quality and the ICP success rate were improved.

Six gold wires were required for the connections between the gold poles of the shell package and the MEMS structures: Two wires for connections of the excitation coils, two for connections of the capacitor plates, and the remaining two for connections of the torsional pendulums fabricated from the low-resistivity silicon.

There were four gold pillars located at both sides of the tube for bonding. However, there was little Kovar alloy present except for the pins. If the magnetic sensor was well placed for the measurement of a horizontal magnetic field, both the interference and shielding of the external magnetic field caused by the gold pillars could be ignored.

**Figure 5:**
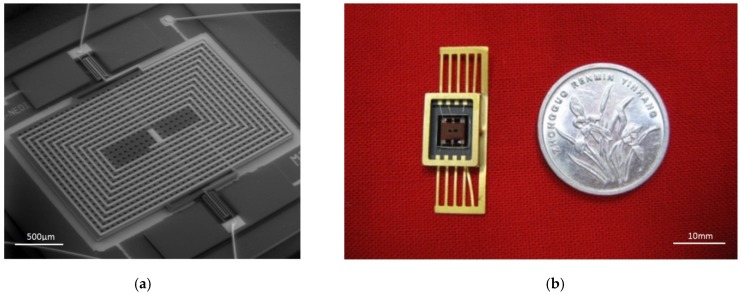
(**a**) MEMS structures for the torsional resonant magnetometer; (**b**) prototype of the MEMS torsional resonant magnetometer.

The MEMS structure and the packaged torsional resonant magnetometer are shown in Figure 5a,b.

## 4. Driving and Detection Circuit

The driving and detection circuit of this MEMS torsional resonant magnetometer included two printed circuit boards (PCBs), connected with a pin header. The bottom PCB was a signal carrier generator, which could generate a very stable and accurate carrier signal on the MHz level. The upper PCB was the miniature magnetometer and a circuit for the signal preprocessing, including a driving circuit, a pre-amplifier circuit, a subsequent amplifier circuit, and a full wave rectifier and demodulator circuit.

**Figure 6:**
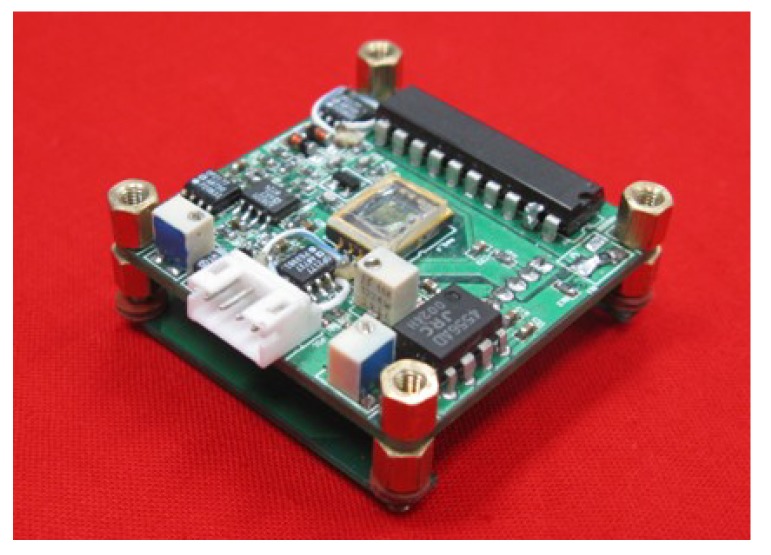
Integrated driving circuit and signal conditioning circuit of the MEMS torsional resonant magnetometer.

The upper PCB had a four-layer structure, where the internal power layer and the Ground (GND) layer were in the middle. Meanwhile, a large number of GND wires were placed on the top and bottom layers to reduce the environmental noises introduced to the pre-amplifier circuit. Because the radiations of the high-frequency carrier signals will cause interferences with other circuits, and the wire of the high-frequency carrier signal is directly connected to the structure of the torsional pendulum, this connecting wire should be as short as possible. In this device, the bandwidth was closely related to the quality factor (Q) value. It was about 0.65 Hz in vacuum and 232 Hz in air. Figure 6 is the integrated driving circuit and signal conditioning circuit of the MEMS torsional resonant magnetometer.

## 5. Testing and Analysis

In this magnetometer, the beam length was 500 μm, and the beam width was 20 μm. The thickness of the resonator was 60 μm. The area of the torsional pendulum structure was about 3000 μm × 2000 μm. The torsional beam parameters are shown in Table 1. To investigate how relevant parameters influenced device performance, six different types of magnetometers were designed with different MEMS structures. As presented in Table 1, the differences included the shapes and dimensions of the torsional beams, the number of coil turns, the diameter of the damping holes, and the dimensions of the torsional pendulums.

Here, the difference between the two straight beams was the beam width, *w*, and the differences between the two folded beams were the beam width and the connection length between the main beam and the side beam. There were two types of exciting coil layers, single layer (M1, M2, and M3) and double-layer (M4, M5, and M6). Meanwhile, there were also two different coil arrangements (7 turns with a 50 μm width and 11 turns with a 30 μm width) for each type. 

Except for M3, the size of the torsional pendulums was 3000 μm × 2000 μm. For the structural limitation, the samples with the narrow excitation coils had 864 damping holes with a diameter of 30 μm, and the samples with the wider excitation coils had 364 damping holes with a diameter of 50 μm.

### 5.1. Sensitivity

**Figure 7:**
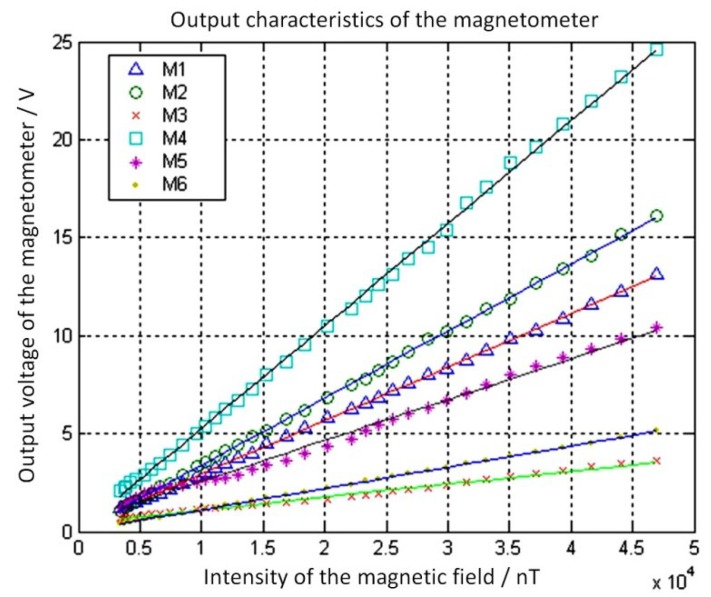
Output properties for different magnetometer models. Reproduced with permission from [29], published by IEEE, 2013.

The output properties of the different magnetometer models were tested in a magnetic field intensity range of 0–50 μT with an energizing current amplitude of 1 mA and a vacuum of 10 Pa at room temperature. The test results are shown in Figure 7. For simplicity, the measured voltage was the peak-to-peak AC voltage signal without synchronous demodulation, which had the same frequency as the driving signal.

For the different magnetometer types, it was clear that there was a very good linear correlation between the output voltage and the magnetic field intensity. In contrast, their sensitivity differences were obvious. As shown in Table 2, the sensitivity of the M4 model was about 8 times that of the M6 model. This was due to the different MEMS structures and parameters, including the torsional beam structures, torsional beam parameters, coil types, torsional pendulum sizes, and the parameters and quantities of the damping holes.

### 5.2. Resonant Frequency and Quality Factor

**Figure 8:**
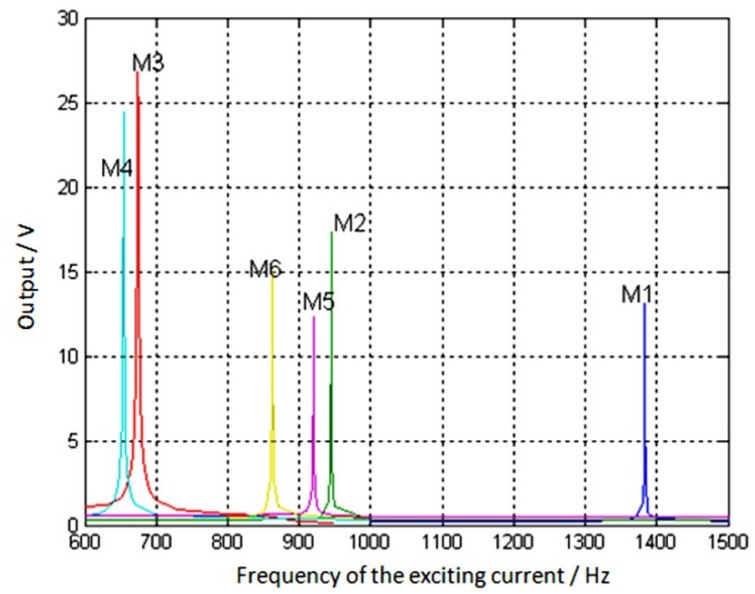
Frequency characteristics of the exciting current for different samples. Reproduced with permission from [29], published by IEEE, 2013.

With a constant outside magnetic field strength and varying excitation current frequency, amplitude changes were observed in the magnetometer output voltages. The frequency characteristics of the exciting current for the different samples are shown in Figure 8. 

Because the vacuum level in the test environment was 10 Pa, the damping ratio, *α*, of the magnetometer torsional pendulum resonant system was close to 0. Therefore, the quality factor, *Q*, could be obtained as follows:

(3) $$Q=f_{0}/\left(f_{u}-f_{d}\right),$$

where *f_0_* is the resonant frequency of the torsional pendulum, and *f_u_* and *f_d_* are the upper and lower frequency limits, respectively, when the output amplitude decreases to $\sqrt{2}/2$ (−3 dB) of the peak. 

**Figure 9:**
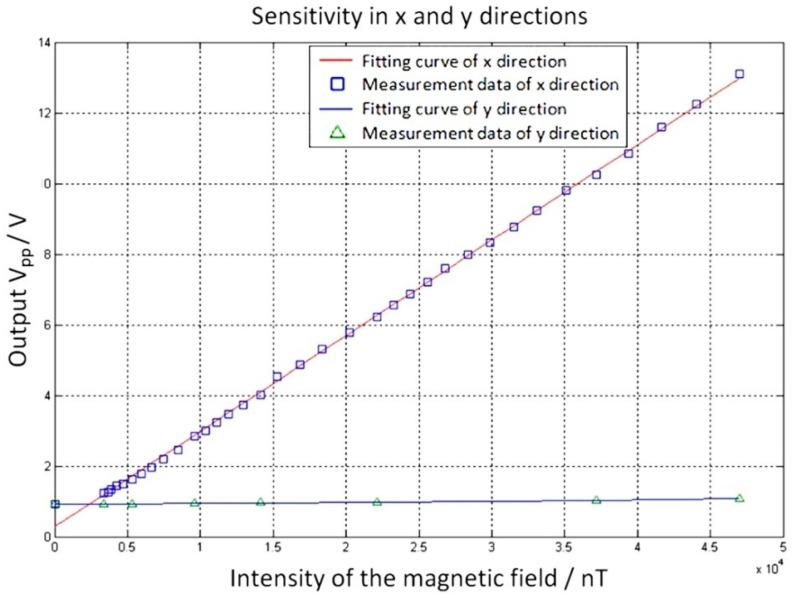
Comparison of the sensitivity in orthogonal axes.

From Equation (3) and Figure 9, the resonant frequency and the quality factor for each sample were obtained. As shown in Table 3, the resonant frequency test results were generally in agreement with the theoretical analysis, and the error was about 15%.

### 5.3. Analysis of the Structural Parameters

The parameters of different magnetometer types were analyzed, and their performance was compared to verify the parameter choices.

#### 5.3.1. Resistance of the Excitation Coil

Ten samples of each type were selected, and the coil resistance was measured. The results showed that the resistance of the double coils was twice that of a single coil with the same number of turns and width. As the upper and lower coils were approximately the same length, it was reasonable that the resistance of the double coil layer was approximately twice that of the single coil layer. This indicates that the fabrication based on the novel design was successful. There were no short circuiting phenomena between the upper and the lower coils due to low fabrication quality of the SiNx insulating layer. According to the measurement results, the double coil process resulted in a high yield. In Table S3, we list the measured and calculated coil resistances.

#### 5.3.2. Initial Value of the Capacitor Polar Plate

The initial capacitor value, C0, between the torsional structure and the capacitor polar plate beneath was simulated by CoventorWare, resulting in a value of 2.663 pF. It was also measured with a capacitance–voltage (C–V) characteristic tester, with results ranging from 2.6 pF to 2.7 pF, which was in good agreement with the simulation result.

#### 5.3.3. Elastic Coefficient of the Torsional Beam

When other structural parameters remained the same, the sensitivity of the M1-type device with a straight beam (width of 25 µm) was 272 mV/µT, whereas the M2-type device with the folded beam (main beam width of 30µm, side beam width of 30 µm) was 342 mV/µT. The sensitivity ratio was thus $K_{2}/K_{1}\approx 1.26$.

The simulation analysis showed that the capacitance variance of M1 was $\Delta C_{1}=2.94\;\mathrm{fF}$, while that of M2 was $\Delta C_{2}=4.15\;\mathrm{fF}$ with an exciting current of 30 mA and a magnetic field intensity of 50 µT. For this type of magnetometer, the ratio of the capacitance variance amplitudes, the ratio of the magnetometer output voltage amplitudes, and the ratio of the sensitivities were the same. Based on the simulation results, the sensitivity ratio of these two devices was $\Delta C_{2}/\Delta C_{1}\approx 1.41$, which agreed well with the experimental data. In addition, it can be seen that the sensitivity of the folded beam device was 1.3 times that of the straight beam device.

Because the excitation coil was led out along the torsional beam, the excitation coil and the torsional beam had the same width. However, the equivalent width of the folded beam was different from that of the straight beam, making their allowable currents different. According to previous experience with the MEMS process, the maximum allowable current of a gold coil with a sectional size of 1 µm × 1 µm was 4 mA, which was proportional to the coil width and thickness. Due to the difference in the equivalent width, the maximum allowable current ratio of the folded beam type and the straight beam type device was approximately 1.2 (30/25). The current amplitude was approximately proportional to the amplitude of the capacitance variance under small angle torsion. Additionally, the sensitivity of the folded beam type was approximately 1.6 (1.3 × 1.2) times that of the straight beam type at the maximum allowable current. Thus, the sensitivity was clearly improved with the folded beam structure.

For comparison of straight beam-type devices with different widths (M1 and M4), from Equation (1), $\omega =\sqrt{k/\Theta }$ was obtained. The moment of inertia (*Θ*) was the same for the two devices, and the elastic coefficient of a straight beam, *k*, was proportional to $w^{3}\gamma$, where *w* and *γ* are the width of the straight beam and a coefficient related to *w*/*h*, respectively. For the M1 device,w/h≈0.417, and γ was 0.254. For the M4 device,w/h≈0.25, and γ was 0.282 [30]. Therefore, the theoretical resonance frequency ratio between M1 and M4 could be obtained as follows:

(4) $$\frac{f_{1}}{f_{4}}=\frac{\omega_{1}}{\omega_{4}}=\sqrt{\frac{k_{1}}{k_{4}}}=\sqrt{\frac{w_{1}^{3}\gamma_{1}}{w_{4}^{3}\gamma_{4}}}=\sqrt{\frac{25^{3}\times 0.254}{15^{3}\times 0.282}}\approx 2.04$$

The practical frequency ratio of the two devices was $f_{c1}/f_{c4}=1383.2/654.4\approx 2.11$, which agreed well with the theoretical results. According to Equation (3), the elastic coefficient of the torsional beam was proportional to the cube of the beam width. Therefore, decreasing the width could notably decrease the elastic coefficient of the torsional beam, thus increasing the torsional angle under the same torque.

#### 5.3.4. Layer Quantity of the Exciting Coil

According to the analysis, the torque generated by the magnetic sensor was the sum of the torques generated by each turn of the exciting coil perpendicular to the magnetic field. In the double coil structure, the number of exciting coils in that direction was twice that of the single coil structure. Therefore, the torque amplitude, *M*, was theoretically twice that of the single coil structure. The maximal twisting angle of the magnetometer torsional pendulum could be calculated as follows:

(5) $$\varphi_{r}=\frac{M}{k}Q$$

where *Q* is the quality factor. Analysis of the performance of the capacitance measurement circuit showed that the amplitude of the output voltage was directly proportional to the capacitance variance amplitude, Δ*C*, and thus was also directly proportional to the maximal twisting angle, *φ_r_*, of the torsional pendulum when the twisting angle was small. The M1-type device had a single exciting coil with a torsion beam width of *w*_1_ = 25 µm, *Q* = 2350.1, and sensitivity = 272 mV/μT. The M4-type device had a double exciting coil with a torsion beam width *w*_4_ = 15 µm, *Q* = 485.9, and sensitivity = 523 mV/μT. Other than these previously mentioned differences, these two device types had the same structures. The structural parameters and test data for the M1- and M4-type devices were input into Equation (4) to obtain the ratio between the torques of the M4 exciting coil and the M1 exciting coil as follows:

(6) $$\frac{M_{4}}{M_{1}}=\frac{\varphi_{r4}Q_{1}k_{4}}{\varphi_{r1}Q_{4}k_{1}}=\frac{K_{4}Q_{1}k_{4}}{K_{1}Q_{4}k_{1}}=\frac{523\times 2350.1\times 15^{3}\times 0.282}{272\times 485.9\times 25^{3}\times 0.254}\approx 2.23$$

According to the theoretical analysis, the expected torque ratio between the M4-type and M1-type devices was 2, which was basically consistent with the test value of 2.23. This indicates that the design of the double exciting coil structure was effective, and the increased excitation torque of the device was approximately double the original value. Analysis of the device linearity showed that its sensitivity was also double the original value.

### 5.4. Sensitivity in the Orthogonal Axis

This magnetometer could only sense the magnetic field in the horizontal plane perpendicular to the torsion beam (the *x* axis in Figure 1). To investigate its sensitivity in the orthogonal axis, the sensitivity was tested in the *x* and *y* directions. From Figure 9, the sensitivity along the *x* axis and *y* axis was *K_x_* = 270 mV/µT and *K_y_* = 3.25 mV/µT. The sensitivity in the *x* direction was about 83 times that in the *y* direction. Therefore, the sensor could be regarded as a monoaxial sensitive sensor.

### 5.5. Repeatability

**Figure 10:**
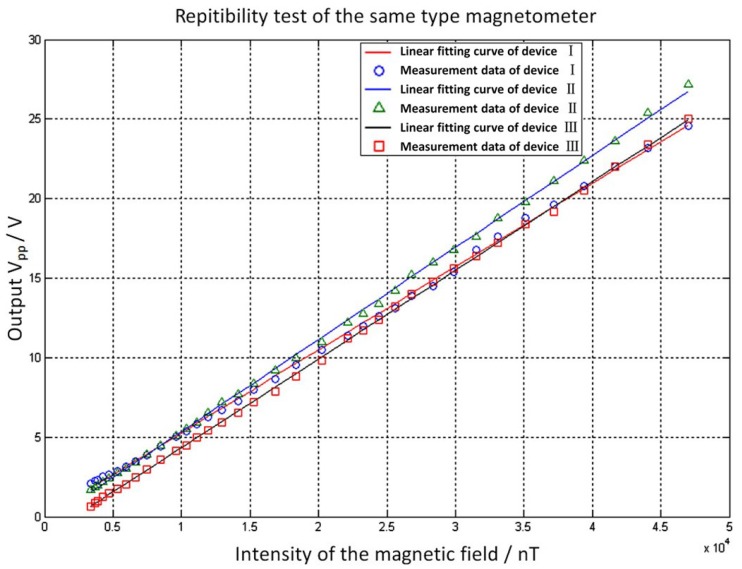
Linear fitting curves for measurement data of the micro magnetometer.

The M3-type device was selected to test the repeatability of the output characteristics. With a pressure intensity of *P* = 5 Pa, an excitation voltage frequency of *f*_0_ = 673.9 Hz, and an excitation voltage peak value of *V*_pp_ = 300 mV, the intensity of the external magnetic field was adjusted three times from 0 nT to 30,000 nT to investigate the change in output voltage with each adjustment of the magnetic field intensity. Linear fitting was performed for each of the three measurements, and the resulting curves are shown in Figure 10.

The performances reflected by the linear fitting curves for the three measurements are summarized in Table 4. The linear correlation coefficient of the data and the sensitivity of the fitting curve were very close, indicating good repeatability.

Next, the main causes of repeatability error were analyzed when the magnetometer was placed in a magnetic field with a variable external magnetic field intensity. As some of the components on the circuit board and the package shells contained nickel, iron, cobalt, and the Kovar alloy compounded from such materials, these components were prone to magnetization and would generate a static magnetic field of a given intensity. When the strength of the magnetic field changed, these materials were also magnetized with an altered strength, and the influence of the magnetic field they generated would also vary. This caused the sensor to output different values for a magnetic field with the same strength during different measurements, thus decreasing the repeatability of the sensor.

**Figure 11:**
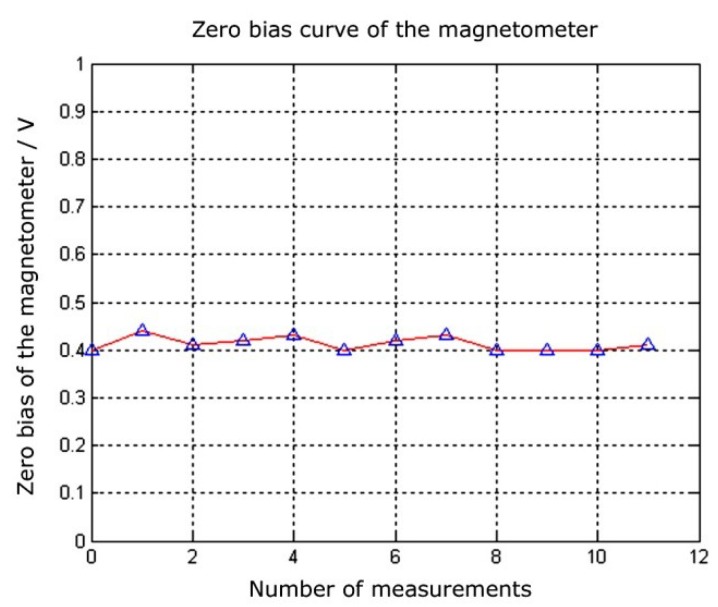
Testing curve for the zero deviations of the prototype.

Meanwhile, during multiple measuring processes with the same magnetometer, the zero deviations of the prototype were recorded for each measurement. The resulting curve is shown in Figure 11. The zero deviations of the prototype ranged between 0.40 V and 0.44 V, with an average of 0.413 V. The standard deviation was approximately 14 mV. This standard deviation was very small relative to the output amplitudes, which satisfied the repeatability requirement.

### 5.6. Noise

**Figure 12:**
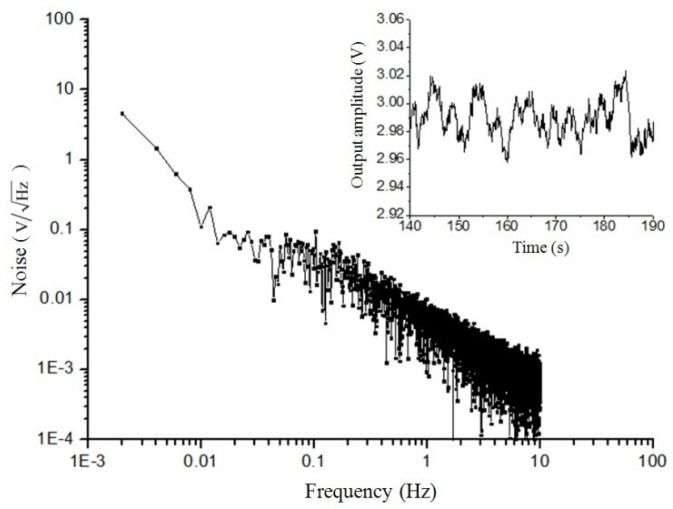
Test result of the noise power spectral density.

Noise was one of the most important parameters of the sensor. Figure 12 shows the noise spectrum of the sensor. The inset shows a typical time trace. Its noise peak-to-peak value was 60 mV. Since the sensitivity of the sample in this range was 1 V/μT, the noise was $60\;\mathrm{nT}/\sqrt{\mathrm{Hz}}$.

## 6. Conclusions

A MEMS torsional resonant magnetometer was designed based on the Lorentz force. MEMS processes were analyzed and optimized based on fabrication experiments and tests. Detailed tests were conducted on the prototypes to verify the choice of design parameters. Experimental measurements showed that the capacitance change could reach 100 fF under the condition of a 10 Pa air pressure when an external magnetic field of 50 μT was applied. Such sensitivity effectively decreased the difficulties in the signal processing. Experiments indicated that the resolution of the device was better than 30 nT. Additional tests demonstrated good repeatability of the measurement process as well as the zero deviations of the device.

To increase the sensitivity, the drive current *I*, coil length *L*, pendulum width *W*, and beam length *l* should be maximized. At the same time, the plate distance *d*_0_, beam thickness *h*, beam width *w*, and shear modulus *G* should be minimized. The quality factor should be as high as possible, which can be realized by increasing the size and the number of holes.

Compared to other MEMS magnetometers, the structures of this torsional resonant magnetometer were easier to fabricate. The test results demonstrated that it could meet the requirements of attitude determination systems of low earth orbit satellites.

## Figures and Tables

**Table 1:** Structural parameters of different magnetometer samples.

Sample Number	Torsional Beam	Coil Layers	Coil Turns	Coil Width (μm)	Damping Hole Diameter (μm)	Damping Hole Quantity
M1	Straight-I	Single	10	30	30	864
M2	Folded-I	Single	10	30	30	864
M3	Folded-II	Single	6	50	50	364
M4	Straight-II	Double	10	30	30	864
M5	Folded-I	Double	6	50	50	308
M6	Folded-II	Double	6	50	50	308

**Table 2:** Sensitivity of different device types.

Sample Number	Analytic Expression of the Fitting Curve *x* (nT), *y* (V)	Sensitivity
M1	*y* = 0.000272*x* + 0.228	272 mV/µT
M2	*y* = 0.000342*x* – 0.0287	342 mV/µT
M3	*y* = 0.000066*x* + 0.438	66 mV/µT
M4	*y* = 0.000523*x* + 0.0489	523 mV/µT
M5	*y* = 0.000208*x* + 0.499	208 mV/µT
M6	*y* = 0.000108*x* + 0.360	108 mV/µT

**Table 3:** Measured and calculated resonance frequency of different magnetometer samples.

Sample Number	*Q* Value	Calculated Frequency (Hz)	Measured Frequency (Hz)
M1	2530.1	1696	1383.2
M2	1684.5	1047	944.8
M3	317.0	798	674.3
M4	485.9	868	654.4
M5	1132.6	1021	919.9
M6	1038.5	970	862.4

**Table 4:** Results of the three measurements.

Serial Number	Linearly Dependent Coefficient *r*	Sensitivity *K* (mV/µT)	Analytical Expression *x* (nT), *y* (V)
1	0.9970	461	*y* = 0.000461*x* + 0.0294
2	0.9939	454	*y* = 0.000454*x* + 0.0359
3	0.9962	469	*y* = 0.000469*x* + 0.00319

## Supplementary Materials

The following are available online at http://www.mdpi.com/2072-666X/9/12/666/s1, Figure S1: The layout of the double coil. (a) The upper coil of the double coil system, and (b) the sublayer of the double coil, connecting the upper layer coil in the center, Figure S2: Circuit principle of the magnetometer, Figure S3: Sketch map of the torsional structure, Table S1: Torsional beam parameters, Table S2: Comparisons of different prototypes, Table S3: Measured and calculated coil resistances of different magnetometer samples.
