# Supplementary material for: A Miniature Resonant and Torsional Magnetometer Based on Lorentz Force
Source: Micromachines (Basel). 2018 Dec 17;9(12):666. doi: 10.3390/mi9120666 (PMC6316825; doi:10.3390/mi9120666)
Supplement: Supplementary file 1 [file micromachines-09-00666-s001.pdf]

A Miniature Resonant and Torsional Magnetometer Based on Lorentz Force

Supplementary Material 1: Layout of the Double Coil System

Figure S1 is the layout of the double coil. The left one is the upper coil of the double coil system, and the right one is the sublayer of the double coil, connecting the upper layer coil in the center.

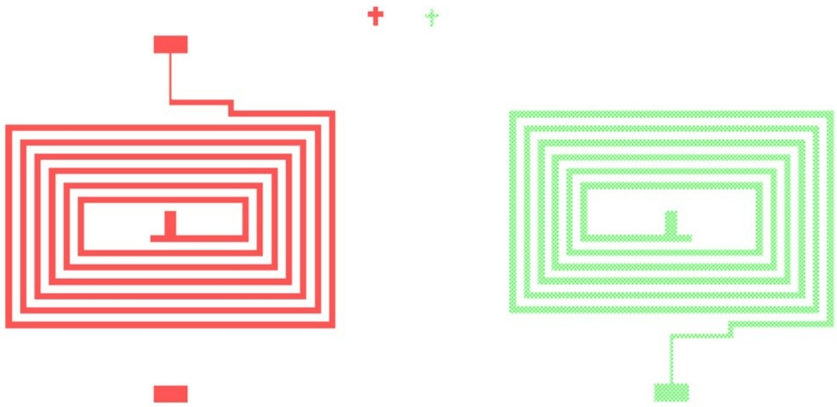

(a) (b)

Figure S1. The layout of the double coil. (**a**) The upper coil of the double coil system, and (**b**) the sublayer of the double coil, connecting the upper layer coil in the center.

Supplementary Material 2: Electronic Circuit Design

The main principle of the electronic circuit is shown in Figure S2.

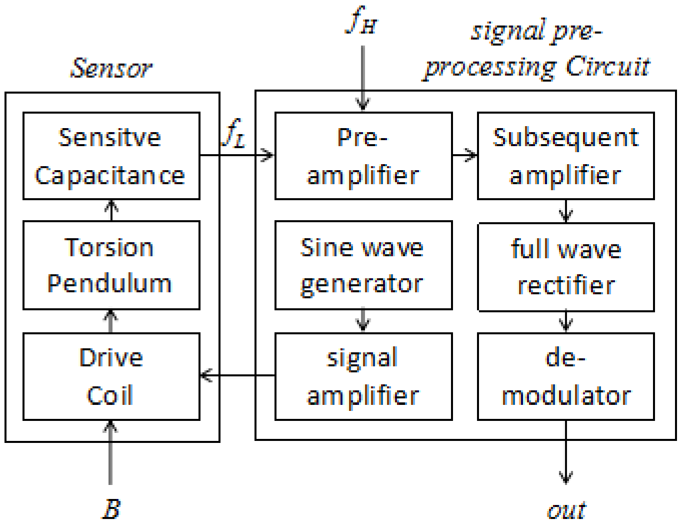

Figure S2. Circuit principle of the magnetometer.

Supplementary Material 3: Torsional Beam Parameters

There were differences in the torsional beam parameters, which are shown in Table S1.

Table S1. Torsional beam parameters.

Beam Type	Length	Thickness	Width
Straight-I	500 µm	60 µm	25 µm
Straight-II	500 µm	60 µm	15 µm
Folded-I	1350 µm	60 µm	main, 30 µm<br>side, 15 µm
Folded-II	1350 µm	60 µm	main, 25 µm<br>side, 15 µm

Supplementary Material 4: Analyses of Different Prototypes

The sensitivity could be explained by the functions below:

$$S = S_M S_\varphi S_{\Delta C}, \quad (S1)$$

$$S_M = \frac{\partial M}{\partial B_x} = ILW, \quad (S2)$$

$$S_\varphi = \frac{\partial \varphi}{\partial M} = \frac{1}{\sqrt{(k-\theta\Omega^2)^2 + c^2\Omega^2}} \approx \frac{Q}{2k} = \frac{Ql}{2Ghw^3\left[\frac{1}{3} - \frac{64}{\pi^5}\frac{w}{h}\sum_{n=1,3,5,\infty}^{\infty}\left(\tanh\frac{n\pi h}{2w}\Big/ n^5\right)\right]} = \frac{Ql}{2Ghw^5\gamma}, \quad (S3)$$

$$S_{\Delta C} = 2\frac{\partial \Delta C}{\partial \varphi} = 2\left[\frac{\varepsilon_0 L}{sin^2\varphi} ln\frac{d_0 - \frac{W}{2}tan\varphi}{d_0} + \frac{\varepsilon_0 LW}{\left(d_0 - \frac{W}{2}tan\varphi\right)sin2\varphi}\right]. \quad (S4)$$

S: Transfer function from magnetic flux density to capacitance variation
S_M: Transfer function from magnetic flux density to torque
S_φ: Transfer function from torque to deflection angle
$S_{\Delta C}$: Transfer function from deflection angle to capacitance variation
I: Current
k: Torsion elastic coefficient
θ: The moment of inertia
Ω: Drive frequency
c: Damped coefficient
G: Shear modulus
γ: $\gamma = \frac{1}{3} - \frac{64}{\pi^5}\frac{w}{h}\sum_{n=1,3,5,\infty}^{\infty}\left(\tanh\frac{n\pi h}{2w}\Big/ n^5\right)$
ε_0: Permittivity of vacuum
L, W, h, w, l, d_0: Structure sizes

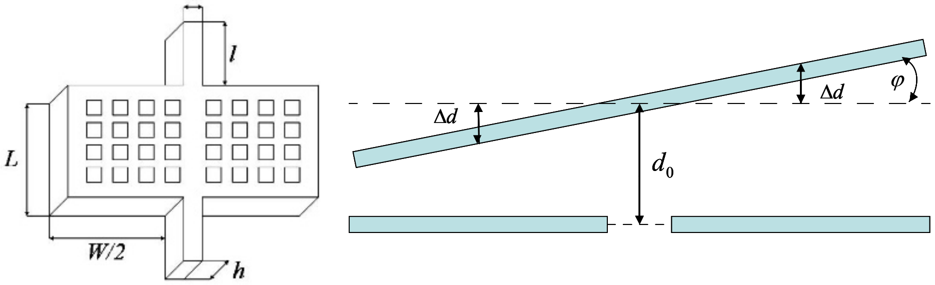

Figure S3. Sketch map of the torsional structure.

Table S2. Comparisons of different prototypes.

Sample number	M1	M2	M3	M4	M5	M6
S	1	1.1	1.08	25.6	1.32	1.8

From the sensitivity function above, M1 was used as a normalization standard. It can be seen that M4 was the best design.

Supplementary Material 5: Measured and Calculated Coil Resistances

Table S3. Measured and calculated coil resistances of different magnetometer samples.

Sample Number	1	2	3	4	5	6	7	8	9	Calculated
M1	103	103	100	103	96	103	102	110	110	128
M2	109	105	120	137	106	103	107	100	96	128
M3	63	57	56	54	55	52	52	51	52	46
M4	158	268	219	295	292	273	266	281	260	256
M5	120	25	112	117	84	116	117	75	104	92
M6	126	113	112	130	112	117	115	96	117	92
